# Apprentices’ Attitudes Toward Using a Mental Health Mobile App to Support Healthy Coping: Mixed Methods Study

**DOI:** 10.2196/35661

**Published:** 2022-08-18

**Authors:** Isabella Choi, Katherine Petrie, Rochelle Einboden, Daniel Collins, Rose Ryan, David Johnston, Samuel B Harvey, Nicholas Glozier, Alexis Wray, Mark Deady

**Affiliations:** 1 Central Clinical School Faculty of Medicine and Health University of Sydney Sydney Australia; 2 Black Dog Institute Faculty of Medicine University of New South Wales Sydney Australia; 3 Susan Wakil School of Nursing and Midwifery Faculty of Medicine and Health University of Sydney Sydney Australia; 4 School of Nursing Faculty of Heath Sciences University of Ottawa Ottawa, ON Canada; 5 WorkSafe ACT Canberra Australia

**Keywords:** apprentice, coping strategies, mental health, app, wellbeing, focus group, coping behaviour

## Abstract

**Background:**

Apprenticeships are a common pathway for young people transitioning into the workforce. Apprentices often face many employment-related challenges and have high levels of psychological distress, drug and alcohol use, and suicidal ideation. Little is known about the attitudes of apprentices toward using smartphone apps to support their mental health and the content that would engage them.

**Objective:**

This study explored (1) apprentices’ interest in using an app to support their mental health and (2) the healthy coping strategies used to manage their mental well-being in the face of workplace challenges, in order to inform future app content.

**Methods:**

A mixed methods study was conducted with 54 apprentices (50/54 male, 93%) with a mean age of 22.7 (SD 5.7) years. Participants completed a survey on preferred ways of using an app to support mental health. Across 8 focus groups, participants were asked to describe healthy strategies they used to cope with occupational stressors.

**Results:**

Only 11% (6/54) of participants currently used a well-being app, but there was high interest in using an app to support their friends (47/54 participants, 87%) and develop self-help strategies to manage or prevent mental health issues (42/54 participants, 78%). Four major types of coping behaviors were identified: (1) social connection for disclosure, advice, and socializing; (2) pleasurable activities, such as engaging in hobbies, time-outs, and developing work-life separation; (3) cognitive approaches, including defusing from thoughts and cognitive reframing; and (4) self-care approaches, including exercise, a healthy diet, and getting adequate sleep.

**Conclusions:**

There is interest among apprentices to use an app with a positive well-being focus that helps them to develop self-management skills and support their friends. Apprentices utilized a range of healthy behaviors to cope with workplace stressors that can be incorporated into mental health apps to improve uptake and engagement. However, many of the preferred coping strategies identified are not those focused on by currently available apps, indicating the need for more targeted digital interventions for this group.

## Introduction

Young people aged 16 to 24 have the highest prevalence of mental disorders but are the least likely to use mental health services [1]. Apprenticeships are a common pathway for young people making the transition from adolescence to adulthood and the working life. In Australia, there were approximately 400,000 individuals commencing apprenticeships or in apprenticeship training in 2020, with about 70% aged under 25 [2]. Men are more likely to enter into apprenticeships than women, with the construction, electrical, and metals and vehicle industries being the largest trade apprenticeship areas [3]. Workers employed in these male-dominated industries or occupations (eg, construction, transport and utilities, mining, and manufacturing) are at higher risk than the general population for psychological distress [4], depression [5], alcohol- and drug-related harms [6], and suicide [7]. Young apprentices face the additional challenges of unrealistic expectations, long work hours, job insecurity, workplace hierarchies, and bullying, which can impact their mental health and well-being [8]. Apprentices have higher levels of psychological distress and drug and alcohol use than national population norms [9,10]. Almost a third of construction apprentices experienced suicidal ideation in the previous year, which is significantly higher than the national norms for young people aged 16 to 25 years [11].

The small body of Australian research mainly focuses on apprentices in the commercial cookery and construction industries and suggests that apprentices tend to manage their work-related stress through maladaptive coping strategies. Two-thirds of apprentices consumed alcohol at harmful levels [12], and a quarter of apprentices reported they had used cannabis in the previous month [9]. A recent study found that apprentices used a range of stress management strategies, such as hobbies and exercise, alcohol and drug use, and taking stress home to their partners and families [13]. Their employment-related stress also contributes to apprentices not completing their training, with about half of trade apprentices in Australia dropping out, and many doing so within the first year of training [14]. While there is a lack of research on help-seeking by apprentices, it is known that those employed in male-dominated occupations are less likely to seek help from professional sources than those in other occupations [15]. They are more likely to adhere to traditional masculine norms [15], in which help-seeking is seen as a sign of weakness, loss of control, and incompetence; stoicism and self-reliance are preferred [16,17].

Smartphone apps may be more acceptable to apprentices as an accessible tool to self-manage their work stress and mental health. Apart from general advantages, such as privacy and anonymity, ease of access, and immediacy, digital mental health interventions are more acceptable to young people who prefer self-reliance and have concerns about stigma [18]. Young people in Australia aged 18 to 34 most commonly use mobiles phones to access the internet (97%), many going online multiple times a day; they are quick to adopt different platforms and apps [19]. There is promising evidence that smartphone apps can improve depression and stress among young adults [20]. Recent studies suggest that about 25% of college students [21] and young people during the pandemic [22] were interested in using mental health apps. While many college students perceive mental health apps to be beneficial, some feel they would not personally use them or do not see a use for them [23]. Little is known about the attitudes of apprentices toward using smartphone mental health apps or the content that would engage them.

Taking into account user preferences is important in improving the uptake and engagement of smartphone mental health apps [24]. A review of young people’s preferred features in digital mental health interventions recommended that apps build on the existing interests of young people in nonconfronting ways, have relatable content and aesthetics, and provide opportunities to learn psychological skills to improve well-being without too much educational or patronizing content [25]. These suggestions are similar to those reported by workers in male-dominated industries, who preferred mental health apps that provided quick, solution-focused strategies for fixing problems and avoided using the stigmatized term “mental health” [26]. Young men have also expressed a preference for online programs that are relevant to their everyday lives and interests and focus on action-based strategies [27]. They are more attracted to digital interventions that focus on positive aspects of mental well-being, such as “happiness,” “strength,” and “mental fitness” [28]. This suggests the possibility that a smartphone app focusing on self-help strategies and healthy coping may be more acceptable to apprentices, especially those working in male-dominated industries.

In order to inform the development of a smartphone mental health app that is acceptable and relevant to apprentices, the current study aimed to (1) explore their interest in using an app to support their mental health and (2) explore the healthy coping strategies used by this group to manage their mental well-being in the face of workplace challenges to inform future app content.

## Methods

### Participants and Recruitment

Registered group training organizations in Sydney and Newcastle, Australia, promoted the study to apprentices through their communication channels, which included emails, flyers, newsletters, and intranet notices. The promotional material invited apprentices to take part in a study exploring how to support apprentice mental well-being. Interested participants registered with an onsite training group coordinator. Participants had to be enrolled in an apprenticeship program, be fluent in the English language, and be a resident of Australia.

### Procedure

A mixed methods study was conducted with 54 apprentices from September to November 2017. This study was part of a larger qualitative study exploring the mental health challenges of apprentices in the workplace [8], their healthy coping strategies, and their attitudes toward using an app to support their mental health (the focus of this paper); the larger study also included focus testing of the Headgear smartphone app (a behavioral activation and mindfulness app that was initially designed to improve the mental health of workers in male-dominated industries) [29]. All participants were reimbursed with an Aus $40 (US $27.97) Visa gift card for their time.

#### Survey

Participants completed a brief, anonymous, paper-based survey at the beginning of the focus group, which included demographic items and questions about current app use for well-being. They were asked to nominate their 2 most significant psychosocial stressors at work. They were also asked to rate how interested they were in the following mental health app content: education about mental health, ways to seek help, self-help strategies, and ways to support a mate. Finally, they were asked how interested they were in using an app focusing on the following areas: managing or preventing depression, anxiety, stress, sleep problems, and substance use; finding out about their risk of developing a mental health problem; and improving physical health, with items scored on a 5-point Likert scale from “not at all interested” to “extremely interested,” based on previous work [30].

#### Focus Groups

Eight activity-based focus groups (with between 3 and 11 participants in each session) were conducted by mental health researchers with experience working with young people. Participants provided written consent. Each focus group was conducted by 2 researchers and lasted approximately 90 minutes. A semistructured discussion guide was used to explore the workplace mental health challenges faced by apprentices (as reported by Einboden et al. [8]) and the types of healthy coping strategies they used to manage these challenges, which is the focus of the current paper. Sticky notes were used to capture individual responses to questions related to mental health challenges prior to sharing as a group. Activity-based approaches are useful for accessing views and opinions on sensitive topics and give variety to the discussion, which is especially useful for young people [31].

### Analysis

Consistent with methods for the analysis of generative participatory data [32], the transcripts of audio recordings were collated with the participant-generated artifacts and coded using an inductive approach to thematic analysis [33-35]. The coding was conducted independently by 2 researchers, manually (KP) and using Quirkos software (RE). The researchers compared codes and discussed their findings, reaching consensus on coding structures and common concepts. Themes were generated and refined through discussion over a series of meetings, and then reviewed by the research team [34]. Following this, MD reviewed the recordings and artifacts to provide additional input. Psychosocial stressors reported in the survey were classified into 8 distinct thematic categories. These categories were not established a priori, but instead were guided by the responses provided.

Survey data were analyzed using Statistical Package for the Social Sciences (SPSS) for Windows (version 23.0.0, IBM). Only descriptive data are reported.

### Ethics Approval

This research was approved by the Human Research Ethics Committee at the University of Sydney (2017/648).

## Results

### Participant Characteristics

Participants were predominantly male (93%), with a mean age of 22.7 (SD 5.7) years (range 16 to 42 years). Most were completing their apprenticeship in the Sydney metropolitan area (76%), were in the first or second year of their apprenticeship (83%), and were undertaking the apprenticeship full-time (83%). The groups included apprentices with 7 different specializations, with the majority undertaking an electrical-related, commercial cookery or hospitality, or construction apprenticeship (Table 1).

**Table 1:** Sample characteristics (N=54).

Characteristics		Values
Age (years) mean (SD)		22.7 (5.7)
**Gender, n (%)**		
	Male	50 (93)
	Female	4 (7)
**Type of smartphone owned, n (%)**		
	Android	23 (43)
	iPhone	28 (52)
	Other (Google, Windows)	3 (6)
**Regular use of well-being or health apps, n (%)**		
	No	48 (89)
	Yes	6 (11)
**Length of time in apprenticeship, n (%)**		
	Less than 1 year	15 (28)
	1 to 2 years	30 (56)
	3 to 4 years	8 (15)
	Not reported	1 (2)
**Type of apprenticeship, n (%)**		
	Full-time	45 (83)
	Part-time	7 (13)
	School-based	2 (4)
**Area of study, n (%)**		
	Electrical-related	26 (48)
	Commercial cookery or hospitality	14 (26)
	Construction (electrician, plumber, or bricklayer)	10 (19)
	Not reported or other	4 (7)
**Location of apprenticeship, n (%)**		
	Metropolitan area (Sydney)	41 (76)
	Regional areas (Hunter and Central Coast)	11 (20)
	Not reported	2 (4)

### Survey Findings

#### Attitudes Toward Using an App for Mental Health

Only 6 of the 54 participants (11%) reported regular use of a well-being app. One female participant mentioned Headspace (a commercial mental health app focused on mindfulness meditation) [36], while 5 male participants reported using fitness monitoring apps, such as Apple iOS Health or Garmin. However, apprentices expressed considerable interest in using a smartphone app for managing mental health and as a source of information about mental health issues. The greatest level of interest among apprentices was in finding ways to support a friend, with 87% (47/54) of respondents “moderately, very, or extremely interested” in using their smartphone to discover strategies for this issue. Self-help strategies to manage or prevent mental health issues were also associated with high levels of interest, with 78% (42/54) reporting moderate or greater interest. Education about mental health and ways to seek mental health if needed were the least popular elements, but still saw 69% (37/54) and 72% (39/54) scoring at least moderate interest, respectively.

Respondents were most interested in an app that focused on improving their physical health; 78% (42/54) reported moderate or greater interest. There was moderate interest in an app that offered strategies to reduce anxiety, with 67% (36/54) reporting moderate or greater interest; stress, with 67% (36/54) reporting moderate or greater interest; and depression, with 65% (35/54) reporting moderate or greater interest. There were lower rates of interest in an app to improve substance use, with 57% (31/54) reporting moderate or greater interest, and sleep, with 57% (31/54) reporting moderate or greater interest. Most respondents (38/54, 70%) were moderately, very, or extremely interested in an app that helped identify their risk of future mental health problems.

#### Psychosocial Stressors

A total of 90 individual work-related stressors were reported among the participants. The most commonly reported workplace stressors related to issues around workplace pressures and expectations (38/90, 42%), followed by personal time factors (work-life balance and long hours) (14/90, 16%), workplace bullying and hostility (11/90; 12%), and interpersonal problems with other employees or the public (11/90, 12%). The remaining reported stressors related to study issues (5/90; 6%); anxiety, boredom, and uncertainty (5/90; 6%); a lack of guidance (3/90, 3%); and financial issues (3/90, 3%).

### Focus Group Findings

Overall, 10 key strategies emerged as a means of coping with work stress. These strategies were categorized into four types of healthy coping behaviors: (1) social connection, (2) pleasurable activities, (3) cognitive approaches, and (4) self-care. In addition, active learning, starting to save or make a budget, and substance use were coping strategies that were mentioned infrequently.

#### Social Connection

Social connection was discussed by participants in all focus groups. Social connection was characterized into 2 distinct, but related, forms. First, it was used as a social strategy (disclosure and advice) for coping, particularly for obtaining advice. In this form, “talking” was viewed as an action, that is, a specific avenue to overcome problems and receive reassurance. Second, it was used as a way of spending time and being together (ie, socializing), which places a greater emphasis on the intangible functions of “social” connections (eg, friendship). There was a feedback relationship between these 2 themes that directly impacted the effectiveness of each element (eg, the more trust within a social connection that was built in the latter, the greater the use of that connection in the former).

Within the strategy of disclosure and advice there was significant use of active verbs (eg, “ask,” “talk,” and “discuss”) to off-load and share stress and a tendency toward seeking information from others; representative quotes are shown in parentheses (eg, “Find someone to talk to like family or partner”). Commonly reported sources of support were friends, family, romantic partners, bosses, and coworkers. Trust played a crucial role in the process (eg, “Talk to family, friends, trustworthy co-workers,” “Rant to another chef you know well enough,” “Pull my head chef or other close work colleagues aside”). Overall, this strategy provided 3 main functions: advice-seeking (eg, “Talking to people that can help come up with strategies with expenses, balance, stresses”), learning skills and practical support (eg, “Ask other apprentices for assistance with difficult studies,” “Ask colleagues for help and tips”), and coping with stress or work issues (eg, “Confronting the issue of pay and co-workers,” “Talk about stuff I am struggling with to parents and friends”).

The most common topic was simply the act of “talking,” while other topics included work issues, skills or study, and “off-loading about work.” Participants were reticent to mention mental health or employment-related stress specifically, preferring instead to use vague terms such as “my problems” or “hard stuff” when talking to others.

The socializing theme emphasized aspects of sharing with, being with, and spending time with friends and family, with primary use of passive verbs (eg, “being with,” “see friends,” “hang out with,” “spend time with”). Reiterating the frequent mention of friends in the “time-out” theme (discussed below), friends and romantic partners were the most commonly mentioned connection. The direct role that socializing played in coping was rarely expressed; instead, it seemed that the act of maintaining these connections satisfied an innate need for support and belonging that was essential to coping. There were also clear links to other themes and strategies, particularly disclosure and advice, work-life separation, and hobbies.

#### Pleasurable Activities

Hobbies were discussed as a prominent means of coping. Common hobbies reported were related to music, movies, television, videos, and outdoor activities.

One participant described hobbies as a way to escape negative feelings: “Have a hobby, find something you like and [you] have an escape.” The use of hobbies for escapism was a means of cognitive distraction: “watch movies and videos to get it off your mind.” Related to the idea of distraction was personal enjoyment, in that hobbies, as one participant put it, equated to “Me time,” that is, “Have a hobby—do things away from work that are healthy, and you enjoy.” The ability to devote time freely to one’s own pursuits rather than feeling the external pressure and constraint experienced at work was viewed as an important component in the use and benefits of hobbies. This was discussed specifically in the context of bullying, workplace constraints and authoritarian workplace structures, and the demands of study.

The second strategy (time-out) was related to the idea of escapism but focused on physical or mental distance without the need for a hobby to fill this space. This was achieved in different contexts and places, both at work (eg, “smoko” [Australian slang for a cigarette break or a rest from work], “being on-break”) and away from work in usual surroundings (eg, “Enjoy weekend off work,” “Speak to work and take a day off”) or on holidays (eg, “Go down the coast somewhere or away,” “Just drive”), as well as mental time-outs (eg, “Zoning out at lunch and smoko breaks”).

The third strategy involved work-life separation and described a higher-level goal of this domain: addressing challenges related to working hours and high pressure. This was particularly pronounced within the context of apprenticeships, which require juggling study, long working hours, overtime, and commuting. These all contributed to increasing stress and the demands of work on respondents’ time and thus required a very deliberate and premeditated “separation” in order to switch off: “When I leave work of an evening I turn all notifications (email) off and forget about work-related issues.”

Both hobbies and time-outs functioned as facilitators to work-life balance, but the role of strict “planning” was central to this strategy. There were also links to other domains, such as scheduling in time for friends and family, exercise, and adequate sleep (“Plan out your week so you have a balance of work/social life/and any sports etc.”).

#### Cognitive Approaches

There were 2 strategies discussed that utilized a cognitive approach to coping. The first was widely discussed and focused on cognitive strategies to defuse from thoughts. The second used elements of cognitive reframing that allowed respondents to motivate and challenge themselves, often fostering a “big picture” focus on their aims and goals to provide motivation to support them through day-to-day challenges. Overall, these approaches provided a source of internal support and strengthened self-belief and resilience.

Defusion strategies varied, but included using distraction (eg, “Try not to think about work,” “Distract yourself from work issues when at home”), relaxation and breathing techniques (eg, “Take some deep breaths and refocus myself on the job at hand”) and “worry time” (eg, “Set aside X amount of time a day for worries then move on”).

Reframing strategies sought to refocus thoughts and take perspective. Means to achieve this included focusing on a positive (eg, “Think about the money,” “Knowing and remembering that it is something that I want to do and I love to do”), or an end goal (eg, “Knowing that I have only a few months left to complete,” “[just] finish the apprenticeship”), using humor as stress relief (eg, “Have a joke,” “Find a funny side...”) and practicing acceptance (eg, “More stressing will not change an outcome”).

#### Self-Care Approaches

Exercise and physical fitness were the most frequently discussed self-care strategies. In all groups, there was a consistent theme of pursuing an ideal of masculine strength: “Train[ing] hard to release the beast.” However, the indirect benefits of exercise (enjoyment, tension release, getting outside, and mental fitness) were also discussed. Furthermore, physical fitness was discussed in the context of being “able to perform necessary functions at work,” especially if the respondents were in a physically demanding industry. The most commonly reported forms of physical activity were walking, going to the gym to work out and train, going to the beach or surfing, and sports generally.

Two other strategies that were less commonly discussed were eating a healthy diet and getting adequate sleep. Healthy diet was generally described as “attempting to have a healthy diet,” while often experiencing lapses into unhealthy food consumption (eg, “[you have to] Try to meal prep so you do not eat shit”). The idea of prepreparation and taking meals to work was specifically related to the challenges associated with time management.

The strategy of getting adequate sleep was raised as a mitigating factor against common challenges related to fatigue, early starts, and long hours (eg, “Get enough rest the night before work”). It was mentioned as both an exercise in self-discipline (eg, “Go to bed early enough so I get enough sleep for the next day”) and a reward (eg, “sleeping in [whenever you're able]”).

#### Other Approaches

The following approaches were less frequently mentioned in the focus groups. Active learning was one technique used in order to address the challenges of study and work expectations. Some participants mentioned using proactive approaches to reinforce concepts and knowledge (eg, “[I] practice electronics outside of work,” “[I focus on] getting my assignments correct and passing...and learning more at work”). Similarly, this practical approach to problem solving was also mentioned in the context of financial challenges, collectively termed savings and budgets (eg, “Saving for a certain thing rather than blowing money,” “Make a budget for the week”). Despite being asked specifically to describe “healthy coping strategies” in the interviews, substance use (eg, “Having a drink,” “Drugs,” “Smoking”) was mentioned by one group as a usual activity they engaged in during time-outs.

## Discussion

### Principal Findings

This study aimed to explore the attitudes of apprentices toward using an app to support their mental health and to explore their use of healthy coping strategies to manage their mental well-being in the face of workplace challenges, in order to inform relatable and nonconfronting app content. All participants owned a smartphone, but few had ever used a mental health or well-being app. Most of the male apprentices who had used a well-being app had used a fitness-monitoring app, but none had used an app specifically for their mental health. There was a high level of interest among apprentices in using an app to support their friends or to learn self-help strategies to manage or prevent mental health issues. Consistent with previous research on preferences for mental health app features among young people [25], apprentices were least interested in using the app for education about mental health.

### Apprentice Mental Health App Design Considerations

#### Focus on Positive Aspects of Well-Being

Supporting the idea that apprentices may be more attracted to positive aspects of well-being, and perhaps reflecting a generally healthy sample, there was high interest in an app focusing on physical health and identifying future risk for mental health problems. These findings suggest an app meeting these needs may be more acceptable (and perhaps less threatening and stigmatizing) than one focused on mental illness. Digital intervention developers and researchers looking to engage apprentices may be best served using approaches that are less direct and improve mental health outcomes circuitously, by encouraging positive coping strategies that enhance well-being in general and outcomes such as physical health, work satisfaction, and “supporting a mate.”

Understanding the healthy coping strategies used by apprentices can inform the design of digital interventions with a nonclinical focus to improve uptake and engagement among this group. The apprentices in this study reported using a range of healthy coping strategies to manage occupational stress, including social connection, pleasurable activities, cognitive approaches, and self-care.

#### Social Connectedness and Seeking Advice

Social connection was a key coping strategy mentioned by all focus groups. While the apprentices reported that socializing and sharing activities with friends were a central part of social connection, they also emphasized the importance of talking and seeking emotional support through disclosure of challenging work situations and seeking advice. However, there was a general reluctance to discuss their internal emotional experiences, and they instead focused on the external forces at play. This is consistent with literature indicating that, when experiencing psychological distress, men are more likely to focus on external circumstances than the emotional experience itself [37]. These results suggest the focus in apps for apprentices should be on encouraging social support and seeking advice, rather than emotional disclosure. Further, apps that build in elements of social support may also facilitate engagement [38], especially among this group.

#### Behavioral Strategies and Self-Care

Many current digital mental health interventions have a cognitive therapy base, whereas the participants in this study have indicated that behavioral strategies (social connection, pleasurable activities, and self-care) form a large part of their healthy coping behaviors. Being able to switch off from work, whether through engaging in a hobby, exercise, time-out activities, or practical strategies such as turning off emails, was a key part of managing their stress at work. This suggests the need for more targeted, action-oriented approaches to engage this group. Mental health apps developed for male-dominated industries and young men with a focus on behavioral activation [39] and positive psychology and social connection [40] have shown promising results. Healthy diet and regular sleep were less commonly discussed, and their importance, with strategies to improve physical health, could be further emphasized in apps for this group.

#### Practical Psychological Skills

Notwithstanding the results described above, the use of cognitive strategies among participants suggests that many cognitive behavioral therapy and mindfulness approaches may still play an important role among apprentices. Defusion strategies were commonly mentioned, which suggests a use and preference for more practical and action-based strategies (eg, calm breathing and worry time) among this group. Participants also reported some use of reframing strategies, such as focusing on positives or end goals. This suggests there is room to introduce value-driven goal setting as part of behavioral activation to reconnect apprentices to an environment of positive reinforcement and improve well-being.

#### Short, Action-Based App Activities

Our sample of apprentices reported that time pressure, workload, and long hours were key workplace stressors. While apps are generally well-placed to support those who are short on time, activities offered in apps also need to be of appropriate length, easily integrated into the daily lives of apprentices, offered in different modalities, and customizable to facilitate engagement [38]. For example, an appropriate activity might be a 2-minute breathing exercise or a value-driven activity planning exercise that apprentices can practice during breaks or after work.

### Limitations

There are several limitations to this study that should be considered. There was a high representation of male apprentices in this sample, the apprentices were recruited from a limited number of male-dominated industries, and they were completing their apprenticeships primarily in the Sydney metropolitan area. The issues faced by and coping strategies used by this cohort may not be representative of all apprentices, especially those working in other trades, industries, or geographic areas, or by female apprentices. Most of the participants were in their late teens or early twenties, though there were a very small number of apprentices who were over 30 years old, so the findings may be less relevant to mature-age apprentices. Participants knew that this study was focused on mental health and well-being, so it is likely there was a bias toward those who were more comfortable discussing these issues. The study did not examine the mental health status of participants, so it is unclear whether their personal experiences of mental health affected their choice of coping strategies or their attitudes to an app to support mental health. While apprentices were asked about their smartphone and well-being app use, we did not explore other aspects of digital literacy in this study. Finally, expectations of a mental health app and preferred features were not directly explored during the focus groups, but were instead explored during the user-testing phase of the Headgear app [29].

### Conclusions

Although many evidence-based smartphone mental health apps exist, most focus on mental health problems, such as depression, anxiety, or distress [20,41]. They do not cater to the user preferences and needs of apprentices, as evidenced by our finding that only one apprentice reporting having used a mental health app. Given that apprentices have shown a preference for apps with a positive well-being focus that helps them to develop self-management skills, our team has adapted a behavioral activation and mindfulness-based smartphone app (Headgear) for apprentices. Headgear provides a risk-profiling tool and a tailored 30-day mental health challenge that includes psychoeducational videos; mindfulness exercises; value-driven activity planning, goal setting and review; and coping skill development (problem solving, sleep, grounding, alcohol use, assertiveness, and training in adaptive forms of coping). In a large-scale randomized controlled trial, the app was found to reduce depression symptoms and prevent incident depression caseness [39]. Adaptations for apprentices included minor modifications to personalization of the risk-profiling tool, altered wording to increase accessibility, the addition of an orientation video, improved navigation, specific apprentice support service guidance, the ability to skip through certain challenges, and elements to enhance gamification (including badges for achievements). A pilot trial of the app showed promising uptake, good engagement, and good acceptability among apprentices, though a full-scale efficacy trial is still needed [29].

The current findings indicate that there is interest among apprentices in male-dominated industries in using an app to support their mental health. Further, there is scope to develop smartphone apps for apprentices with a well-being focus by incorporating healthy coping strategies, including social connection, behavioral strategies and self-care, and practical psychological skills, which may be seen as more relevant and acceptable ways to support mental health among this population. A mental well-being app targeting the needs of apprentices, such as by helping them learn how to support friends or use short, action-based self-management activities, may be a way to engage apprentices in developing these healthy coping skills and improve their well-being.
